# Laparoscopic Adrenalectomy for Pheochromocytoma in a Child

**Published:** 2013-01-01

**Authors:** Fahimeh Soheilipour, Abdolreza Pazouki, Sahar Ghorbanpour, Zeinab Tamannaie

**Affiliations:** Assistant Professor of Pediatric Endocrinology and Metabolism, Minimally Invasive Surgery Research Center, Rasoul Akram Hospital, Tehran University of Medical Sciences, Tehran, Iran.; Assistant professor of Minimally Invasive Surgery, Minimally Invasive Surgery Research Center, Rasoul Akram Hospital, Tehran University of Medical Sciences, Tehran, Iran.; Medical Student, Member of Medical Students Research Committee, Rasoul Akram Hospital, Tehran University of Medical Sciences, Tehran, Iran.; General Practitioner, Minimally Invasive Surgery Research Center, Rasoul Akram Hospital, Tehran University of Medical Sciences, Tehran, Iran.

**Keywords:** Adrenalectomy, Laparoscopy, Pheochromocytoma

## Abstract

Pheochromocytoma is a catecholamine-secreting tumor of the adrenal medulla. It has wide and subtle range of clinical manifestations including sustained hypertension in about 1% of pediatric patients. Although laparoscopic adrenalectomy is the gold standard treatment method in adult patients, few reports have described this technique in children. We report a child with unilateral pheochromocytoma who presented with poor weight gain, polyuria and polydipsia. Diagnosis was based upon clinical and laboratory evaluation. She was treated successfully by laparoscopic adrenalectomy.

## INTRODUCTION

Pheochromocytoma is a rare tumor in children [1]. The most common manifestation is triad of headache, sweating and palpitations along with hypertension; however, atypical clinical features may be present [2]. Early diagnosis and surgical removal is desirable because the tumor may prove fatal [3]. Ganger et al reported first laparoscopic adrenalectomy in 1992 [3, 4]. About 109 pediatric patients have been treated by laparoscopic adrenalectomy and reported in the world literature [5-11]. Herein, we report laparoscopic adrenalectomy in a child with pheochromocytoma.

## CASE REPORT

A 10-year-old girl presented with failure to thrive despite good appetite for 3 years. She also had a history of polyuria and polydipsia for one year. She was incidentally found to have a blood pressure of 170/110 mmHg. Her body weight was 21 kg (50th percentile for 7 years old), height was 128 cm (10th percentile), and her bone age was about 7 year. Her pulse was 120/min and rest of physical and systemic examinations were unremarkable. A 24-hour urine analysis revealed urine vanillylmandelic acid (VMA) of 25.4 mg/24hours (normal range, 0.4-0.6), metanephrine over 960 mcg/24hours (normal range, 0-350), and normetanephrine over 3840 mcg/24hours (normal range, 0-600). Her serum aldosterone and cortisol levels were normal. Other laboratory tests were within normal limits. Abdominal ultrasonography revealed solid heterogeneous mass with micro cysts, measuring 68 x 58 mm at the left adrenal region. CT scan of the abdomen showed a solid hetero-density mass lesion, measuring approximately 60 mm in diameter, near the upper pole of the left kidney (Fig. 1). Echocardiogram and ECG were unremarkable. 

**Figure:**
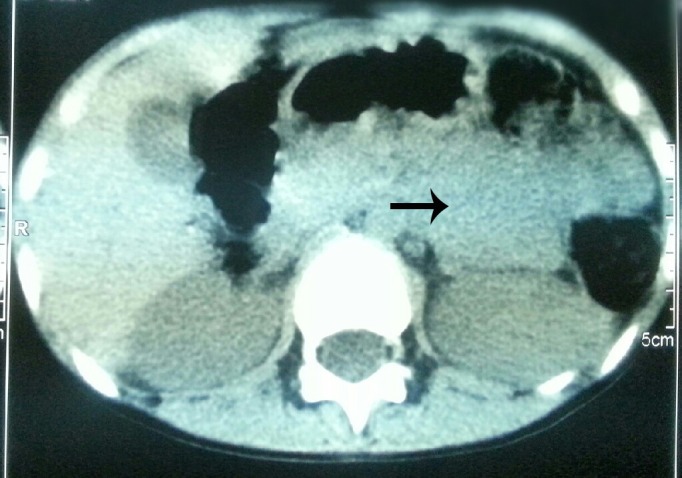
Figure 1: CT scan showing suprarenal mass (arrow).

She was given phenoxybenzamine (alpha blocker) for 14 days preoperatively; starting with 2.5 mg 12 hourly to 10 mg three times a day for resistant hypertension. Patient was also given propranolol (beta blocker) 5 mg four times daily starting from 11th day of preoperative preparation for 4 days. Intravenous hydration was given 24 hours prior to the surgery. After stabilizing her blood pressure and other vital signs she underwent laparoscopic left adrenalectomy (Fig. 2).

**Figure:**
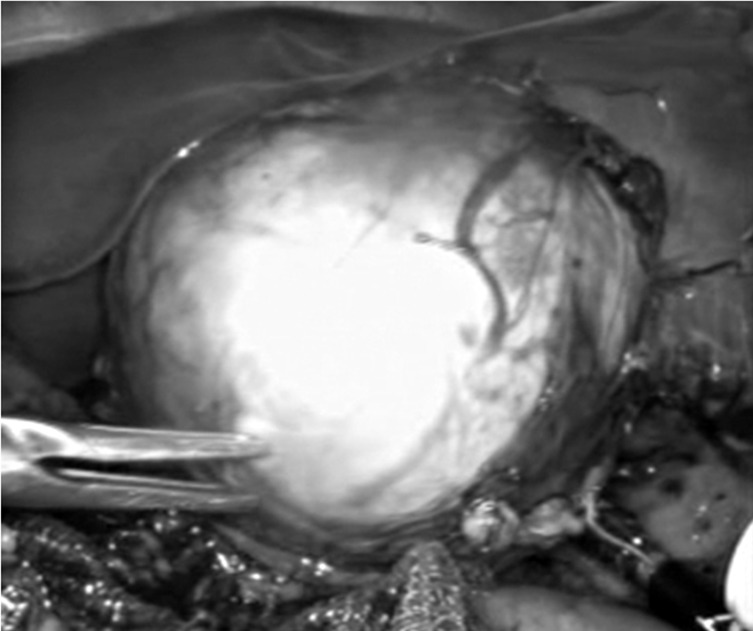
Figure 2: Laparoscopic view of adrenal mass.

The child was placed in dorsal decubitus slightly turned to her right side at a 45° angle. Insufflation was performed through a 10 mm port that was inserted under direct vision at mid clavicular line about 1.5 cm bellow costal margin. A 30° telescope was inserted and, under visual control, three other ports were inserted including one 10 mm (1.5 cm bellow costal margin) and two 5 mm ports (one of them in xiphoid region and one in posterior axillary line 1.5 cm bellow costal margin). The Gerota’s fascia was incised by a 5 mm ligasure. The adrenal gland was released from the spleen, pancreatic tail, and left kidney. Left adrenal vein was exposed and secured with clips and the excised lesion removed in an endoscopic retrieval bag through the 10-mm trocar. A drain was positioned bellow the spleen. All trocars were removed and the wounds closed. The operation time was about 90 minutes. The excised mass was a 30gram encapsulated creamy-gray soft mass measuring 6x5x3.5 cm. Pathological examination of the tumor showed features of pheochromocytoma.

The postoperative period was uneventful. Her blood pressure became normal (120/90 mm Hg) without any medication. 24-hour urine studies were repeated which showed normal level of VMA, metanephrine and normetanephrine. The patient was discharged on postoperative day 4 tolerating a regular diet. At twenty months postoperative follow up, she remained in excellent condition with no clinical or imaging signs of recurrence and her growth index increased significantly.

## DISCUSSION

Pheochromocytoma is a catecholamine secreting tumor that arises from chromaffin cells of the sympathetic nervous system (adrenal medulla and sympathetic chain); however, the tumor may develop anywhere in the body [2]. Pediatric pheochromocytoma is an extremely uncommon and fewer than 5% of these tumors occur in the pediatric age group. Approximately 10% of pediatric pheochromocytomas are familial. Pheochromocytomas are often found on the right side and are sporadic, unilateral, and intra-adrenal [5, 6]. 

The best treatment of adrenal pheochromocytoma is early venous control with radical excision and removal of any local invasion [9]. With the advances in minimally surgery, laparoscopic adrenalectomy is rapidly replacing open adrenalectomy in adult patients. In children, laparoscopic adrenalectomy is also recommended. Adrenalectomy is particularly well suited for the laparoscopic approach because of the relative small size of the adrenal gland, the low incidence of malignancy and the retroperitoneal location [10]. We hope that laparoscopic adrenalectomy be considered in all pediatric patients where indicated.

## Footnotes

**Source of Support:** Nil

**Conflict of Interest:** None declared
